# The Role of Sentinel Lymph Node Biopsy and Factors Associated with Invasion in Extensive DCIS of the Breast Treated by Mastectomy: The Cinnamome Prospective Multicenter Study

**DOI:** 10.1245/s10434-015-4476-5

**Published:** 2015-03-17

**Authors:** Christine Tunon-de-Lara, Marie Pierre Chauvet, Marie Christine Baranzelli, Marc Baron, Jean Piquenot, Guillaume Le-Bouédec, Fréderique Penault-Llorca, Jean-Rémi Garbay, Jérôme Blanchot, Joëlle Mollard, Véronique Maisongrosse, Simone Mathoulin-Pélissier, Gaëtan MacGrogan

**Affiliations:** 1 Department of Surgery, Institut Bergonié, Comprehensive Cancer Centre, Bordeaux, France; 2 Department of Surgery, Centre Oscar Lambret, Lille, France; 3 Department of Pathology, Centre Oscar Lambret, Lille, France; 4 Department of Surgery, Centre Henri Becquerel, Rouen, France; 5 Department of Pathology, Centre Henri Becquerel, Rouen, France; 6 Department of Surgery, Centre Jean Perrin, Clermont-Ferrand Cedex 1, France; 7 Department of Pathology, Centre Jean Perrin, Clermont-Ferrand Cedex 1, France; 8 Department of Surgery, Institut Gustave Roussy, Villejuif, France; 9 Department of Surgery, Centre Eugène Marquis, Rennes, France; 10 Department of Surgery, Centre Hospitalier Universitaire de Limoges, Limoges, France; 11 Department of Pathology, Centre Claudius Regaud, Toulouse, France; 12 University of Bordeaux, Bordeaux, France; 13 Clinical and Epidemiological Research Unit, Institut Bergonié, Comprehensive Cancer Centre, Bordeaux, France; 14 INSERM U897, CIC-EC07, Institut Bergonié, Comprehensive Cancer Centre, Bordeaux, France; 15 Department of Biopathology, Institut Bergonié, Comprehensive Cancer Centre, Bordeaux, France

## Abstract

**Background:**

When invasive components are discovered at mastectomy for vacuum-assisted biopsy (VAB)-diagnosed ductal carcinoma in situ (DCIS), the only option available is axillary lymph node dissection (ALND). The primary aim of this prospective multicenter trial was to determine the benefit of performing upfront sentinel lymph node (SLN) biopsy for these patients. The secondary aim was to determine DCIS factors associated with microinvasion or invasion.

**Methods:**

The SLN procedure was performed during mastectomy, and for positive SLN an ALND was performed during the same intervention. A tissue microarray containing DCIS lesions from the mastectomy specimens was subsequently performed.

**Results:**

From May 2008 to December 2010, 228 patients were enrolled from 14 French cancer centers, including 192 eligible patients with pure DCIS on VAB and successful SLN procedures. ALND was avoided for 51 [67 %; 95 % confidence interval (CI), 56–77 %] of all the patients who had microinvasive DCIS or DCIS associated with invasive carcinoma at mastectomy and a negative SLN. Of the 192 patients, 76 (39 %) with VAB-diagnosed DCIS were upgraded after mastectomy to micro (*n* = 20) or invasive disease (*n* = 56). The rate of positive SLN for patients with DCIS on VAB was 14 %. High nuclear grade of DCIS was associated with greater risk of microinvasion and invasion, and HER2-amplified DCIS was associated with greater risk of invasion.

**Conclusions:**

Underestimation of invasive components is high when DCIS is diagnosed by VAB in patients undergoing mastectomy. Upfront SLN for patients with VAB-diagnosed extensive DCIS avoids unnecessary ALND for two-thirds of patients with micro or invasive disease on mastectomy.

**Electronic supplementary material:**

The online version of this article (doi:10.1245/s10434-015-4476-5) contains supplementary material, which is available to authorized users.

The incidence of ductal carcinoma in situ (DCIS) has dramatically increased, reaching 14.6 % in France in 2009^1^ with the use of breast cancer screening programs. Although mastectomy was the common treatment in the past,^2^ breast-conserving surgery currently is used successfully. Extensive DCIS is not considered in this category, and patients often are recommended to undergo mastectomy. Vacuum-assisted (VAB) biopsies frequently underestimate invasion, with 5–20 % of VAB-diagnosed DCIS cases upstaged to microinvasion or invasive carcinoma at the final pathologic assessment.

Although the indication for sentinel lymph node (SLN) biopsy in the DCIS setting is controversial, it is advised for patients treated by mastectomy or when invasive disease is suspected.^3^ However, no prospective clinical study has ever assessed the pertinence of this strategy.

The SLN biopsy is a minimally invasive procedure, and when results are negative, axillary lymph node dissection (ALND),^4^,^5^ associated with higher morbidity rates,^6^,^7^ can be avoided. When results are positive, ALND remains the standard of care, but its use often has been called into question. The ACOSOG Z0011 trial, for instance, has shown that secondary ALND is not necessary for patients with invasive carcinoma treated by conservative surgery and presenting with fewer than two positive SLNs.^8^ Because mastectomy alters the lymphatic drainage of the breast, axillary staging with SLN biopsy in the weeks after surgery is no longer accurate, and ALND should be performed systematically.^4^

This prospective multicenter study examined the relevance of using the SLN procedure upfront for patients with extensive microcalcifications on mammography and treated by mastectomy. The primary end point was the rate of needless ALND avoided for patients whose mastectomy specimen showed DCIS with microinvasion (DCIS–MI) or DCIS with invasive carcinoma (DCIS–IDC). The secondary end points were the rate of underestimation of invasion by VAB, the discrepancy between extension of microcalcifications on mammography and DCIS histologic size, the rates of SLN detection and positive SLN, and the identification of specific pathologic and immunohistochemical factors of DCIS associated with microinvasion and invasion.

## Methods

### Patients

Patients were recruited from 14 participating French comprehensive cancer centers. These patients were older than 18 years and presented with extensive microcalcifications or multicentric foci (in two different quadrants) of the breast classified as American College of Radiology Breast Imaging-Reporting and Data System (ACR BI-RADS) categories four or five on mammography and a diagnosis of DCIS or DCIS–MI on VAB. The patients had an indication for mastectomy jointly determined by a radiologist and a surgeon because conservative treatment was not feasible. Patients with lumpectomy-diagnosed DCIS or DCIS–MI, previous ipsilateral radiation therapy or ALND, previous in situ or invasive ipsilateral breast carcinoma, or an indication for conservative breast surgery were excluded from this study.

Mastectomy with an SLN procedure was performed as described by Rodier et al.^9^ For each SLN detected, an intraoperative evaluation of frozen sections was performed. For patients with positive SLN, an ALND was performed during the same intervention.

This study was approved by the Committee of Protection of Individuals, Aquitaine, France and performed in accordance with the Helsinki Declaration. All the participants provided written informed consent (clinical trials NCT01841749).

### Pathologic Analysis

The participating centers used a standardized protocol for handling SLN. Fresh, nonfixed SLNs were sent from the operating room to the pathology laboratory for macroscopic analysis. For grossly suspicious SLN, an intraoperative microscopic frozen section analysis was performed. Otherwise, the SLNs were fixed, then grossly sectioned at 2-mm intervals and paraffin-embedded in their entirety.

Each formalin-fixed paraffin-embedded SLN block was sectioned at three levels separated by 300 μm. For each level, a hematoxylin and eosin (H&E) section and three unstained slides were prepared. An immunohistochemical analysis with a cytokeratin antibody was performed only if suspicious nondeterminate cells were found on the H&E section of the SLN.

The mastectomy specimens were X-rayed and drawn on a centimeter grid to determine the correlation between the initial mammographic findings and the histologic analyses. Pathologic sampling was performed using the grid as a template, and tissue blocks containing DCIS were reported on the grid. The pathologic extent of DCIS then was measured directly on the grid. The pathologist evaluated the extent of the DCIS by radiography of the mastectomy specimen.

For verification purposes, the mastectomy specimens were X-rayed and drawn on a centimeter grid for estimation of DCIS size by measurement of the distance between the two furthermost blocks with DCIS involvement. The presence of scar tissue corresponding to the previous biopsy site was searched in every case.

After completion of the study, a central pathology review and a tissue microarray (TMA) were performed with all mastectomy specimens. Other specific pathologic criteria including the presence of necrosis, the nuclear grade within the DCIS lesion, and the presence of a lymphoplasmacytic infiltrate (inflammation) surrounding the DCIS lesion were centrally assessed by a single pathologist (G.M.G.). Pretherapeutic macrobiopsies were not available for central pathologic review.

### Immunohistochemical and Dual In Situ Hybridization Analysis

Immunohistochemical analysis was performed on a Ventana Benchmark Ultra automat (Meylan, France). Supplementary Table S1 summarizes the technical conditions and the antibodies used. Immunohistochemical staining was estimated on the luminal cells in the mastectomy specimens, either in the nuclei for ER, PR, FOXA1, and Ki-67; on the cytoplasmic membrane for HER2, EGFR, and E-cadherin; or in the cytoplasm for CK5/6, CK14, P16, and CSTA. For E-cadherin, a continuous cytoplasmic membrane staining was considered as positive. Any other type was considered negative. The threshold of positivity was 10 % for ER, PR, and FOXA1 and 15 % for Ki-67. For assessment of Ki-67, a semiquantitative method was used, in which the proportion of Ki-67-positive DCIS cells in the overall DCIS cell population was estimated on one histologic section regardless of the number of ducts involved. The HER2 immunostaining was interpreted according to the American Society of Clinical Oncology (ASCO) scoring system applied to DCIS.^10^ The number of positive DCIS cells per tissue section was determined semiquantitatively from 0 to 100 % as well as the intensity of staining for E-cadherin, EGFR, P16, and CSTA. A staining score of 0–300 was obtained by multiplying the percentage of positive DCIS cells by their staining intensity. A threshold of 100 was chosen to separate the positive EGFR, P16, and CSTA cases from the negative ones, and a threshold of 200 was chosen for E-cadherin. The presence or absence of any staining for CK5/6 or CK14 was scored respectively as positive or negative.

The epithelial membrane antigen (EMA) staining pattern in the luminal cells of the DCIS lesions was recorded in accordance with the classification of de Roos et al.^11^ Predominant diffuse cytoplasmic (CD), focal cytoplasmic (CF), diffuse membranous (MD), and apical membranous (MA) patterns of EMA staining were briefly specified. Scoring of COX2 staining was performed according to Kerlikowske et al.^12^ Dual ISH using the Ventana Inform HER2 dual ISH was performed on a Ventana Benchmark Ultra automat. In this study, DCIS was considered as HER2-amplified when the absolute HER2 gene copy number was 6 or higher and the HER2/CEN17 ratio was 2.2 or higher. All cases were analyzed for HER2 status by dual ISH irrespective of their immunohistologic status.

### Statistical Analysis

The primary end point of this study was the rate of ALND avoided in patients with microinvasive DCIS or DCIS associated with invasive carcinoma diagnosed in the mastectomy specimen and the SLNs void of cancer. We calculated the rate as the number of patients with negative SLNs divided by the total number of patients with mDCIS–MI or mDCIS–IDC.

To calculate the required number of patients, we predicted a 10 % underestimation of invasion on VAB. Of this 10 % requiring upstaging, approximately 80 % should have negative SLNs. The rate of avoided ALND was thus estimated to be about 8 % of the patients with DCIS and an indication of mastectomy, and 100 patients were necessary to obtain a corresponding 95 % confidence interval (CI) of about 3.5–15.2 %.

The rate of discordance between VAB and mastectomy was calculated by dividing the number of patients with discordant results between VAB and surgery by the total number of patients. The association between the extension of microcalcifications shown on mammography and the histologic size of DCIS in the mastectomy specimens was analyzed using Spearman’s test.

Univariate analyses using *χ* ^2^, Fisher’s exact test, or Wilcoxon’s rank sum test identified pretreatment radiologic and postmastectomy pathologic and immunohistochemical factors associated with microinvasion and invasion in the mastectomy specimen. All factors significant at a *p* value lower than 0.15 were included in a multiple logistic regression model adjusted for age with a stepwise manual process. Precisely, the following factors and categories were assessed: DCIS radiologic and pathologic factors (histologic size, continuous), nuclear grade (low, intermediate, or high), necrosis (yes vs no), and inflammation (yes vs no), as well as immunohistochemical factors (ER, PR, and FOXA1) (<10 vs ≥10 %); Ki-67 (<15 vs ≥15 %); HER2 (0 or + vs ++ vs +++); CK5/6 and CK14 (positive vs negative); EGFR, P16, or CSTA (<100 vs ≥100); E-cadherin (<200 vs ≥200), EMA (CD+CF vs MA+MD); COX2 (0–1 vs 2–3); and HER2 gene (amplified vs nonamplified). A *p* value lower than 0.05 was considered statistically significant.

## Results

### Inclusions and Initial VAB

**Fig. 1:**
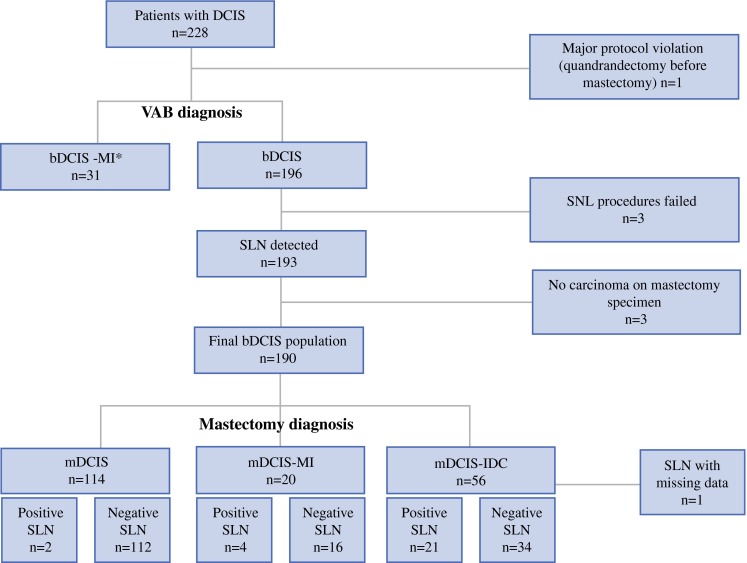
Flow chart of ductal carcinoma in situ (DCIS) patients included in the study and results of the sentinel lymph node (SLN) procedure. *n* number of patients, *ALND* axillary lymph node dissection, *VAB* vacuum-assisted biopsy, *b-DCIS* vacuum-assisted biopsy-diagnosed DCIS, *b-DCIS–MI* vacuum-assisted biopsy-diagnosed DCIS with microinvasion, *mDCIS* mastectomy-diagnosed DCIS, *mDCIS–MI* mastectomy-diagnosed DCIS with microinvasion, *mDCIS–IDC* mastectomy-diagnosed DCIS with associated invasive carcinoma

Between May 2008 and December 2010, 228 women with biopsy-diagnosed DCIS (bDCIS or bDCIS–MI) were included in the study. One major protocol violation was excluded, leaving 227 patients eligible for analysis, including 196 bDCIS and 31 bDCIS–MI patients (Fig. 1). Table 1 presents the presurgical pathologic and radiologic characteristics for the bDCIS after VAB diagnosis. Table 2 shows histologic characteristics from the mastectomy specimen.

**Table 1:** Presurgical pathologic and radiologic characteristics of patients presenting with ductal carcinoma in situ (DCIS) diagnosed on vacuum-assisted biopsy (VAB)

	bDCIS (*n* = 196)
Median age: years (range)	53.4 (24–83)
ACR BI-RADS	
BI-RADS 4	95 (48.5)
BI-RADS 5	96 (49.0)
Missing data^a^	5 (2.5)
Extension of microcalcifications: mm (range)	50 (4–130)
Nuclear grade	
Low	11 (5.6)
Intermediate	49 (25.0)
High	117 (59.7)
Missing data^a^	19 (9.7)
Necrosis	
No	34 (17.4)
Yes	139 (70.9)
Missing data^a^	23 (11.7)
Inflammation	
No	50 (25.5)
Yes	49 (25.0)
Missing data^a^	97 (49.5)

*DCIS* ductal carcinoma in situ, *VAB* vacuum assisted biopsy, *n* number of patients, *ACR BiRADS* American College of Radiology-Breast Imaging-Reporting and Data System

^a^Missing data is the pretherapeutic macrobiopsy were not available for central pathological review

**Table 2:** Histologic characteristics of mastectomy specimens for ductal carcinoma in situ (DCIS) diagnosed on vacuum-assisted biopsy (VAB) patients

	bDCIS (*n* = 196)
SLN detected	192 (98.5)
Negative SLN	165 (85.9)
Positive SLN	27 (14.1)
1 Positive SLN	20 (74.1)
2 Positive SLNs	6 (22.2)
3 or 4 Positive SLNs	1 (3.7)
Mastectomy histologic results	
pT0	3 (1.5)
pT is (pure mDCIS)	117 (59.7)
pT1 mic (mDCIS–MI)	20 (10.3)
pT ≥ 1a (mDCIS–IDC)	56 (28.5)
mDCIS nuclear grade	
Low	14 (7.1)
Intermediate	72 (36.7)
High	101 (51.6)
Missing	9 (4.6)
Necrosis (mDCIS)	
No	35 (17.9)
Yes	152 (77.5)
Missing	9 (4.6)
Inflammation	
No	43 (22)
Yes	144 (73.4)
Missing	9 (4.6)
DCIS size: mm (range)	69.3 (4–180)
Invasive component (for IDC)	56 (100)
Unifocal	30 (57.7)
Multifocal^a^	22 (23.1)
Missing	4 (19.2)
Histological subtype	56 (100)
IDC	53 (94.6)
Others	3 (5.4)
Size (invasive): mm (range)	9.3 (1–45)
pT1	50/56 (89.3)
IDC grade	56 (100)
1	15 (21.4)
2	24 (42.9)
3	16 (28.6)
Missing	4 (7.1)

*bDCIS* vacuum-assisted biopsy-diagnosed ductal carcinoma in situ, *VAB* vacuum-assisted biopsy, *n* number of patients, *SLN* sentinel lymph node, *mDCIS* mastectomy-diagnosed ductal carcinoma in situ, *mDCIS-MI* mastectomy-diagnosed ductal carcinoma in situ with microinvasion, *IDC* invasive ductal carcinoma, *mDCIS-IDC* mastectomy-diagnosed ductal carcinoma in situ with associated invasive carcinoma

^a^Multifocal was defined as 2 or more foci of invasive carcinoma separated by at least 2 mm

### Rate of Unnecessary ALND Avoided

The SLN procedure was successful in identifying SLNs in all but three cases (98 %), and no carcinoma was detected in the mastectomy specimen for three patients, giving a final population of 190 bDCIS patients. Figure 1 shows the results from the histologic analyses. A total of 76 initially pure bDCIS patients were upgraded to micro or invasive events in the mastectomy specimen. Of these patients, 51 had negative SLNs, and an unnecessary ALND was therefore avoided (67 %; 95 % CI, 56–77 %). Of the 25 patients with SLN involvement, 15 underwent ALND [1 isolated tumor cell (ITC), 4 micrometastases, and 10 macrometastases]. In 10 cases (5 ITCs, 4 micrometastases, and 1 macrometastasis), ALND was not performed. These cases involved false-negative frozen section SLN results, and local tumor boards decided not to perform subsequent axillary clearance for clinical or patient preference reasons (Table 3).

**Table 3:** Needless axillary lymph node dissection (ALND) avoided in mDCIS–MI and mDCIS–IDC cases upgraded after mastectomy

Histologic results on mastectomy	bDCIS (*n* = 190)^a^			
	mDCIS (*n* = 114)	mDCIS–MI (*n* = 20)	mDCIS–IDC (*n* = 56)^a^	ALND avoided in mDCIS–MI or mDCIS–IDC
SLN status				
Negative	112	16	35	51/51
Positive	2	4	21^a^	10/25^b^
ITC (≤0.2 mm)	2	1	5	5/6
Micrometastasis (0.2 ≤ 2 mm)	0	1	7	4/8
Macrometastasis (>2 mm)	0	2	9	1/11

^a^1 SLN had missing data

^b^ALND not performed

*bDCIS* vacuum-assisted biopsy-diagnosed ductal carcinoma in situ, *n* number of patients, *SLN* sentinel lymph node, *mDCIS* mastectomy-diagnosed ductal carcinoma in situ, *mDCIS-MI* mastectomy-diagnosed ductal carcinoma in situ with microinvasion, *IDC* invasive ductal carcinoma, mDCIS-IDC mastectomy-diagnosed ductal carcinoma in situ with associated invasive carcinoma, *ITC* isolated tumour cell

### VAB Mastectomy Discrepancy Rate

Figure 1 illustrates the discrepancy between VAB and mastectomy diagnoses. As shown, 39 % (76/196; 95 % CI, 45.8–32.1 %) of the patients with a diagnosis of bDCIS on VAB were subsequently upgraded and, excluding the failed SLN procedures (3/196) as well as the patients with missing SLN data (1/196), the rate of positive SLN was 13 % (25/192).

A correlation was found between the extension of microcalcifications on mammography and the histologic size of DCIS in the mastectomy specimens (*ρ* = 0.215; *p* = 0.005, Spearman’s test).

### Uni- and Multivariate Analyses of Pathologic and Immunohistochemical Factors of DCIS with Microinvasion or Invasion in the Mastectomy Specimen

Pathologic and immunohistochemical factors of DCIS associated with microinvasion in the univariate analyses included the presence of inflammation, ER-negative status, PR-negative status, the presence of necrosis, high nuclear grade, a P16 score of 100 or higher, and a CSTA score of 100 or higher (Table 4). Only high nuclear grade remained a significant independent factor in the multivariable model.

**Table 4:** Uni- and multivariate analyses of pathologic and immunohistochemical factors of ductal carcinoma in situ (DCIS) with concurrent microinvasion (DCIS–MI) and invasive carcinoma (DCIS–IDC) in the mastectomy specimen

	mDCIS–MI			mDCIS–IDC		
	Univariate analysis *p*	Multivariate analysis^a^		Univariate analysis *p*	Multivariate analysis^a^	
		OR (95% CI)	*p*		OR (95 % CI)	*p*
High nuclear grade	0.006	3.1 (1.4–7.0)	0.007	0.12	2.7 (1.3–5.6)	0.008
Inflammation	0.03	NS		0.09	NS	
Necrosis	0.04	NS		0.05	NS	
ER-negative	0.01	NS		NS	NS	
CSTA score ≥100	0.02	NS		NS	NS	
EMA predominant pattern	NS	NS		0.11	NS	
PR-negative	0.096	NS		NS	NS	
P16 score ≥100	0.14	NS		NS	NS	
HER2 amplification	NS	NS		0.009	OR 3.7 (1.7–7.8)	0.001
Ki67	NS	NS		0.05	NS	

*DCIS* ductal carcinoma in situ, *mDCIS-MI* mastectomy-diagnosed ductal carcinoma in situ with microinvasion, *mDCIS-IDC* mastectomy-diagnosed ductal carcinoma in situ with associated invasive carcinoma, *n* number of patients, *SD* standard deviation, *CI* confidence interval, *NS* non-significant

^a^The final multivariate model was adjusted for age

Pathologic and immunohistochemical factors of DCIS associated with invasion in the univariate analyses included the presence of inflammation, the presence of necrosis, high nuclear grade, a predominant EMA membranous staining pattern, amplification of HER2, and Ki-67 of 15 % or higher. In the multivariable model, high nuclear grade and amplification of HER2 were independent factors. Supplementary Tables S2 and S3 provide further details on the uni- and multivariate analyses.

## Discussion

Although standard in the diagnosis of breast and other cancers, SLN biopsy is not justified for all DCIS patients^13^ because DCIS usually is considered noninvasive. Occasionally, an increased risk of invasion exists, and SLN biopsy should be performed to assess the involvement of the axillary nodes. We present a prospective study on the relevance of the SLN procedure for patients who have extensive DCIS with microcalcifications on mammography diagnosed by VAB and treated by mastectomy. We selected three major predictive factors of disease upgrading as patient inclusion criteria:^14^ extensive microcalcifications,^15^,^16^ mastectomy,^4^,^5^,^17^ and VAB diagnosis.^18^^–^ 20

The primary aim was to investigate whether the use of SLN upfront could avoid ALND for patients who have biopsy-diagnosed DCIS with associated micro or invasive carcinoma and negative SLN. We found that 67 % (51/76) of patients presenting with mDCIS–MI or mDCIS–IDC had negative SLNs and avoided complete ALND.

Findings show that SLN status is important when an infiltrative component is associated with the DCIS lesion. Whereas a negative SLN rules out unnecessary ALND, a positive SLN results in a complete ALND. In our study, ALND proved unnecessary for patients presenting with ITC or micrometastases, supporting previous work by Galimberti et al.^21^ However, six nonsentinel nodes were found to be positive in 11 ALNDs performed for patients with macrometastases. These results diverge from those of ACOSOG Z001, which apply to patients treated by breast-conserving surgery followed by whole-breast irradiation therapy.

All the patients in our series were treated by mastectomy without radiotherapy. The low risk of positive SLN usually reported for pure DCIS (0.39–13.7 %)^17^,^20^,^22^^–^ 26 was confirmed by this study, which showed SLN to be positive in 2 % (2/114 ITC) of cases. For mDCIS–IDC and mDCIS–MI, the overall rate of positive SLN was 33 % (25/76) [or 25 % (19/7) excluding ITC], which is significantly higher than the 6.2 % reported by Tada et al.^25^ for invasive carcinoma with extensive DCIS. A direct correlation exists between invasive carcinoma size and SLN positivity.^27^ In our series, the mean size of invasive carcinoma was 9.3 mm (89 % pT1) compared with less than 5 mm in the series by Tada et al.^25^ In three large series, the authors concluded that a microinvasive lesion shown on biopsy or an invasive component shown by surgery significantly increased the risk of positive SLN.^16^,^28^

We observed a high rate of underestimation of invasive components (40 %) compared with rates observed across other VAB-diagnosed series (11.2**–**21 %)^29^^–^ 32 which may have been due to the heterogeneity in the extent of DCIS across series.

In a study comparable with ours, Tan et al.^33^ observed a similar upgrade rate of 33 % for 90 patients with extensive DCIS treated by mastectomy for a DCIS having a mean size of 62 mm. In a recent Canadian study, DCIS size was the only predictor of underestimation, with an odds ratio (OR) of 1.92 (95 % CI, 1.65–2.24) per 1-cm increase in size.^34^ The large size of DCIS lesions in our series (mean size, 69.3 mm) might explain the high rate of underestimation compared with others.^15^,^18^ Indeed, previous results^15^,^16^,^20^ have demonstrated that DCIS size is an independent risk factor for concomitant invasive carcinoma.^16^ Three patients were overtreated with mastectomy because no DCIS was found on the surgical specimen, and DCIS lesions were confirmed after review of the VAB. The other microcalcifications found on the mastectomy specimens were located in benign lesions.

Pathologic and immunohistochemical factors associated with invasive components and DCIS are currently unknown. Previous studies have examined the expression of different markers in DCIS and the risk of subsequent in situ or invasive recurrence.^11^,^12^ We therefore investigated the association between their level of expression in the DCIS lesions and the risk of invasive carcinoma. In univariate analyses, high DCIS nuclear grade, necrosis, and stromal inflammation were associated with both microinvasion and invasion. However, after multivariate modeling, only high nuclear grade was found to be associated with both events. Overexpression of HER2 was an independent predictor of a higher risk of invasive components. Amplification of HER2 in DCIS is more frequent than in invasive carcinoma^35^,^36^ and for some authors may represent a precursor of invasion^37^–^39^ In the NSABP B43 trial, transtuzumab was used as chemopreventive treatment for DCIS with HER2 amplification.^39^

Recently, CSTA, a protease inhibitor of cathepsin B activity, was found to be downregulated in invasive carcinomas adjacent to DCIS.^40^ Surprisingly, we found high levels of CSTA expression in DCIS to be associated with micro invasion, which is somewhat contradictory with the initial finding of Lee et al.^40^ who found that down regulation of CSTA was associated with progression of DCIS to invasive carcinoma. Our findings tend to indicate that DCIS is a heterogeneous pathology that can either remain as pure DCIS or progress to DCIS–MI or DCIS–IDC.

This study confirmed the relevance of the SLN procedure and further encourages recommendation for patients with DCIS diagnosed by VAB presenting with extensive microcalcifications on mammography and treated by mastectomy because it avoids unnecessary ALND for patients with no lymph involvement, ITC, or a single micrometastasis in SLN. Additionally, in terms of staging, SLN biopsy surpasses the accuracy of VAB or mastectomy, with almost four in ten DCIS diagnoses underestimated on the initial VAB and upgraded after mastectomy. Our results also demonstrate that whereas amplification of HER2 is an independent predictor of invasive disease, high nuclear grade is associated with an increased risk of both microinvasion and invasion.

## Electronic supplementary material

Below is the link to the electronic supplementary material.

Supplementary material 1 (DOCX 32 kb)
