# Supplementary material for: The Role of Sentinel Lymph Node Biopsy and Factors Associated with Invasion in Extensive DCIS of the Breast Treated by Mastectomy: The Cinnamome Prospective Multicenter Study
Source: Ann Surg Oncol. 2015 Mar 17;22(12):3853–60. doi: 10.1245/s10434-015-4476-5 (PMC4595535; doi:10.1245/s10434-015-4476-5)
Supplement: Supplementary file 1 — Supplementary material 1 (DOCX 32 kb) [file 10434_2015_4476_MOESM1_ESM.docx]

| **Supplementary Table S1.** Characteristics of immunohistochemical markers used to identify associations between ductal carcinoma in situ with microinvasion or concurrent invasive carcinoma in the mastectomy specimens | | | | |
| --- | --- | --- | --- | --- |
| Antibody | Clone | Dilution | Pretreatment | Incubation time |
| ER | SP1 Ventana | prediluted | ultraCC1 standard | 32 minutes |
| PR | 1E2 Ventana | prediluted | ultraCC1 standard | 32 minutes |
| HER2 | 4B5 Ventana | prediluted | ultraCC1 short | 16 minutes |
| Ki-67 | 30-9 Ventana | prediluted | ultraCC1 standard | 32 minutes |
| EMA | E29 Dako | 1/60 | ultraCC1 standard | 32 minutes |
| P16 | INK4a Ventana | prediluted | ultraCC1 standard | 32 minutes |
| CK5/6 | D5/16 B4 Dako | 1/50 | ultraCC1 short | 32 minutes |
| CK14 | LL002 Diag Biosystems | 1/50 | ultraCC1 short | 40 minutes |
| EGFR | 3C6 Ventana | prediluted | P1 8 minutes | 32 minutes |
| E-cadherin | EP700Y Ventana | prediluted | ultraCC1 standard | 32 minutes |
| FOXA1 | 2F83 AbCam | 1/8000 | ultraCC1 (20 min) | 32 minutes |
| AR | SP107 Ventana-cell Marque | prediluted | ultraCC1 standard | 32 minutes |
| P63 | 4A4 Dako | 1/100 | ultraCC1 standard | 32 minutes |
| COX2 (Dako) | CX-294 | 1/50 | ultraCC1 standard | 52 minutes |
| CSTA | Produced in rabbit (Sigma) | 1/100 | ultraCC1 standard | 56 minutes |
| TRIO | Rabbit  Polyclonal | 1/100 | HIER pH9 (20min on microwave) | Overnight at 4^o^C |

| **Supplementary Table S2.** Univariate analysis of pathological and immunohistochemical factors of DCIS with concurrent micro-invasion in the mastectomy specimen | | | | | | |
| --- | --- | --- | --- | --- | --- | --- |
|  | Diagnosis after mastectomy | | | | | Univariate  analysis |
|  | mDCIS  n=117 | | mDCIS-MI  n= 38 | | |  |
| **n % n %** | | | | | | ***P*** |
| DCIS Size (mm)^*^  Average (SD)  Median [min-max] | 68 (29.6)  70 [4-160] | | | 63.6 (35.4)  60 [4-180] | | 0.33 |
| Nuclear grade  Low and intermediate  High  Missing | 59  52  6 | (50.43)  (44.45)  (5.12) | | 10  27  1 | (26.32)  (71.05) (2.63) | **0.006** |
| Necrosis  No  Yes  Missing | 26  85  6 | (22.23)  (72.65)  (5.12) | | 3  34  1 | (7.89)  (89.48)  (2.63) | **0.042** |
| Inflammation  No  Yes  Missing | 32  79  6 | (27.35) (67.53) (5.12) | | 4  33  1 | (10.53) (86.84) (2.63) | **0.027** |
| ER  <10%  ≥10%  Missing | 32  78  7 | (27.35)  (66.67)  (5.98) | | 19  17  2 | (50)  (44.74)  (5.26) | **0.01** |
| PR  <10%  ≥10%  Missing | 59  51  7 | (50.43)  (43.59)  (5.98) | | 25  11  2 | (65.79)  (28.95)  (5.26) | 0.096 |
| Ki67  <15%  ≥15%  Missing | 28  82  7 | (23.94)  (70.08)  (5.98) | | 6  30  2 | (15.79) (78.9) (5.26) | 0.27 |
| HER2 score  0 or +  ++  +++  Missing | 45  13  52  7  0 | (38.47)  (11.11)  (44.44)  (5.98)  0 | | 12  3  21  2  0 | (31.5) (7.89) (55.2)  (5.26)  0 | 0.51 |
| D-Dish  0  +  NI | 50  58  9 | (42.7)  (49.6)  (7.6) | | 15  21  2 | (39.5) (55.3) (5.2) | 0.63 |
| CK56  Negative  Positive  Missing | 106  5  6 | (90.6)  (4.28)  (5.12) | | 36  1  1 | (94.7) (2.63) (2.63) | 1.0^**^ |
| EGFR  <100  ≥100  Missing | 107  5  5 | (91.46)  (4.27)  (4.27) | | 35  2  1 | (92.11)  (5.26)  (2.63) | 1.0^**^ |
| E-cad score  <200  ≥200  Missing | 27  84  6 | (23.08)  (71.8)  (5.12) | | 9  28  1 | (23.68)  (73.69)  (2.63) | 1.0^**^ |
| COX2  0-1  2-3  Missing | 23  80  14 | (19.66)  (68.38)  (11.96) | | 7  28  3 | (18.42)  (73.69)  (7.89) | 0.77 |
| CSTA score  <100  ≥100  Missing | 76  25  16 | (64.96)  (21.37)  (13.67) | | 19  16  3 | (50)  (42.11)  (7.89) | **0.020** |
| P16 score  <100  100-200  ≥200  Missing | 81  19  6  11 | (69.24)  (16.24)  (5.12)  (9.4) | | 23  9  4  2 | (60.54)  (23.68)  (10.52)  (5.26) | 0.14 |
| EMA predominant pattern  CD+CF  MA+MD  Missing | 77  30  10 | (65.82)  (25.64)  (8.54) | | 30  7  1 | (78.95)  (18.42)  (2.63) | 0.27 |
| ^*^2 Pure DCIS and 1 DCIS-MI missing size  ^**^Fisher’s test used instead of Chi-square  **Abbreviations:** DCIS= ductal carcinoma in situ; mDCIS= mastectomy-diagnosed ductal carcinoma in situ; mDCIS-MI= mastectomy-diagnosed ductal carcinoma in situ with micro-invasion; n= number of patients; SD= standard deviation. | | | | | | |

| **Supplementary Table S3.** Univariate analysis of pathological and immunohistochemical factors of DCIS with concurrent invasive carcinoma in the mastectomy specimen | | | | | | |
| --- | --- | --- | --- | --- | --- | --- |
|  | Diagnosis after mastectomy | | | | | Univariate  analysis |
|  | mDCIS  n=117 | | mDCIS-IDC  n= 69 | | |  |
| **n % n %** | | | | | | ***P*** |
| DCIS Size (mm)^*^  Average (SD)  Median [min-max] | 68 (29.6)  70 [4-160] | | | 68.5 (30.3)  65 [10-140] | | 0.98^‡^ |
| Nuclear grade  Low and intermediate  High  Missing | 59  52  6 | (50.43)  (44.45)  (5.12) | | 28  40  1 | (40.57)  (57.98) (1.45) | **0.12** |
| Necrosis  No  Yes  Missing | 26  85  6 | (22.23)  (72.65)  (5.12) | | 8  60  1 | (11.59)  (86.96)  (1.45) | **0.054** |
| Inflammation  No  Yes  Missing | 32  79  6 | (27.35) (67.53) (5.12) | | 12  56  1 | (17.39) (81.16) (1.45) | **0.091** |
| ER  <10%  ≥10%  Missing | 32  78  7 | (27.35)  (66.67)  (5.98) | | 22  46  1 | (31.88)  (66.67)  (1.45) | 0.64 |
| PR  <10%  ≥10%  Missing | 59  51  7 | (50.43)  (43.59)  (5.98) | | 35  33  1 | (50.73)  (47.82)  (1.45) | 0.77 |
| Ki67  <15%  ≥15%  Missing | 28  82  7 | (23.94)  (70.08)  (5.98) | | 9  59  1 | (13.04) (85.51) (1.45) | **0.051** |
| HER2 score  0 or +  ++  +++  Missing | 45  13  52  7 | (38.47)  (11.11)  (44.44)  (5.98) | | 29  15  24  1 | (42.03) (21.73) (34.79)  (1.45) | 0.12 |
| D-Dish  0  +  NI | 50  58  9 | (42.7)  (49.6)  (7.6) | | 45  23  1 | (39.5) (33.3) (1.45) | **0.009** |
| CK56  Negative  Positive  Missing | 106  5  6 | (90.6)  (4.28)  (5.12) | | 64  4  1 | (92.76) (4.79) (1.45) | 0.73^**^ |
| CK14  Negative  Positive  Missing | 111  1  5 | (94.88)  (0.85)  (4.27) | | 66  2  1 | (95.66)  (2.89)  (1.45) | 0.56^**^ |
| FOXA1  <10%  ≥10%  Missing | 2  109  6 | (1.70)  (93.18)  (5.12) | | 3  65  1 | (4.34)  (94.21)  (1.45) | 0.37^**^ |
| EGFR  <100  ≥100  Missing | 107  5  5 | (91.46)  (4.27)  (4.27) | | 66  2  1 | (95.66)  (2.89)  (1.45) | 0.71^**^ |
| E-cad score  <200  ≥200  Missing | 27  84  6 | (23.08)  (71.8)  (5.12) | | 18  50  1 | (26.08)  (72.47)  (1.45) | 0.74 |
| COX2  0-1  2-3  Missing | 23  80  14 | (19.66)  (68.38)  (11.96) | | 13  51  5 | (36.1)  (38.9)  (7.26) | 0.76 |
| CSTA score  <100  ≥100  Missing | 76  25  16 | (64.96)  (21.37)  (13.67) | | 53  11  5 | (76.80)  (15.94)  (7.26) | 0.25 |
| P16 score  <100  100-200  ≥200  Missing | 81  19  6  11 | (69.24)  (16.24)  (5.12)  (9.4) | | 53  12  3  1 | (76.82)  (17.39)  (4.34)  (1.45) | < 100 *v* ≥ 100  0.81 |
| EMA predominant pattern  CD+CF  MA+MD  Missing | 77  30  10 | (65.82)  (25.64)  (8.54) | | 41  27  1 | (78.95)  (18.42)  (2.63) | 0.11 |
| ^*^2 Pure DCIS and 1 DCIS-MI missing size  ^**^ Fisher’s test used instead of Chi square  ^‡^Wilcoxon’s test  **Abbreviations**: DCIS= ductal carcinoma in situ; mDCIS= mastectomy-diagnosed ductal carcinoma in situ; mDCIS-IDC= mastectomy-diagnosed ductal carcinoma in situ with associated invasive carcinoma; n= number of patients ; SD= standard deviation | | | | | | |
